# An Innate Color Preference Displayed by *Xenopus* Tadpoles Is Persistent and Requires the Tegmentum

**DOI:** 10.3389/fnbeh.2020.00071

**Published:** 2020-05-12

**Authors:** Jasper Elan Hunt, John Rudolph Bruno, Kara Geo Pratt

**Affiliations:** ^1^Department of Zoology and Physiology and Program in Neuroscience, University of Wyoming, Laramie, WY, United States; ^2^Department of Physiology, Anatomy and Genetics, University of Oxford, Oxford, United Kingdom

**Keywords:** innate, color, behavior, *Xenopus* tadpole, tegmentum, trazodone, swimming speed

## Abstract

Many animals, especially those that develop externally, are equipped with innate color preferences that promote survival. For example, *Xenopus* tadpoles are known to phototax most robustly towards mid-spectrum (“green”) wavelengths of light while avoiding shorter (“blue”) wavelengths. The innate preference to phototax towards green likely promotes survival by guiding the tadpoles to green aquatic plants—their source of both food and safety. Here, we characterize the dynamics and circuitry that give rise to this intriguing hard-wired behavior. Using a novel open-field experimental paradigm we found that free-swimming tadpoles indeed spend most of their time in the green portion of the test dish, whether green is pitted against white (brighter than green) or black (darker than green). This preference was modest yet incredibly persistent over time, which, according to the shell game model of predator-prey interactions, minimizes being found by the predator. Furthermore, we found that this innate preference for the color green was experience-independent, and manifested mainly *via* profoundly slower swimming speeds while in the green region of the test dish. Ablation experiments showed that, at the circuit level, the color-guided swimming behavior requires the tegmentum, but not the optic tectum (OT). Lastly, we determined that exposing tadpoles to the selective serotonin reuptake inhibitor (SSRI) trazodone switched the tadpoles’ preference from color-based to luminance-based, implicating two distinct visual circuits in the tadpole, one that is associated with color-driven behaviors, another associated with luminance-driven behaviors.

## Introduction

Throughout the animal kingdom, color vision is important for survival. Animals use color cues for navigation, and to locate food, shelter, mates, and predators. So fundamental are color cues that many color-guided behaviors are not learned but are innate. Innate color preferences have been described in many organisms including zebrafish (Spence et al., 2008; Guggiana-Nilo and Engert, 2016; Park et al., 2016), *Drosophila* (Lazopulo et al., 2019), bees (Ostroverkhova et al., 2018), chickens and ducks (Hess, 1956; Kovach, 1971), hawkmoths (Kuenzinger et al., 2019), and several different species of frogs and tadpoles (Muntz, 1963; Jaeger and Hailman, 1976). Experimentally, innate color-driven behaviors have provided a convenient read-out for studying photoreceptors and the ability of an organism to differentiate between different wavelengths of light. Beyond the level of the retina, much less is understood about the mechanism(s) underlying innate color-driven behaviors (Kelber et al., 2003). The focus of this study is on the neural underpinnings of color-driven behavior itself, in the *Xenopus laevis* tadpole. Many aspects of the *Xenopus laevis* (*X. laevis*) tadpole render it an interesting model organism for studying innate visual behaviors. First, this species of *Xenopus* has existed for approximately 17.5 million years (Furman et al., 2015), whereas other commonly studied amphibians such as *Bufo americanus* and *Ambystoma tigrinum* diverged only 1.3 million years ago (Masta et al., 2002) and 10 million years ago (Shaffer and McKnight, 1996), respectively. Beyond being a highly-conserved species, *Xenopus laevis* is one of the most highly invasive species known, so understanding its behaviors provides insight about its enduring success. Second, a popular model to study neural circuit formation and function, the *Xenopus* tadpole visual system has been described in detail (reviewed in Liu et al., 2016; Pratt et al., 2016). The rods of the *Xenopus* retina are especially interesting in the context of color vision because they contain opsins that are normally found in the different wavelength-tuned cones, yet, like mammalian rods, are still sensitive to low-intensity (i.e., dim) light. This means that tadpoles and frogs can discriminate different colors at their detection thresholds (Solessio et al., 2004; Parker et al., 2010; Yovanovich et al., 2017), advantageous for identifying colors of plants or other animals at the bottom of murky South African ponds. Finally, previous phototactic studies report that tadpoles display innate color preferences, preferring to phototax towards mid-spectrum wavelengths of light (green), and avoiding short (blue) wavelengths (Muntz, 1963; Jaeger and Hailman, 1976). These phototactic studies employ a forced-choice paradigm in which the tadpole is forced to make a one-time decision to phototax towards one of two different colored arms of the test chamber. Here, to extend the findings generated *via* the forced-choice assays, we use a novel open-field test paradigm designed to characterize the long-term color preferences of freely-swimming developmental stage 48/49 tadpoles. By developmental stages 48/49—approximately 11–18 days post fertilization (dpf)—tadpoles display robust visually guided behaviors including visual avoidance behaviors, optomotor responses (OMRs; Dong et al., 2009), and the ability to differentiate between different colors (Blackiston and Levin, 2012; Rothman et al., 2016). Using this approach we identified an enduring color preference, the behavioral dynamics that give rise to the preference, and the underlying circuitry required for its expression.

## Materials and Methods

### Tadpoles

All animal husbandry and experimental procedures were approved by the University of Wyoming’s Institutional Animal Care and Use Committee (IACUC). *Xenopus laevis* tadpoles were obtained from either in-house matings of adult wild-type *Xenopus* frogs or from Nasco (Ft. Atkinson, WI, USA). Tadpoles were raised in Steinberg’s solution and housed on a 12:12 light/dark cycle in white-walled incubators at 22°C. All experiments were performed on developmental stage 48/49 tadpoles (approximately 11–18 dpf; staging per Nieuwkoop and Faber, 1994) that had never been fed. Each experimental group was comprised of data pooled from four or more different clutches. Stages 48/49 encompass over 1 week of the tadpole’s life cycle (Nieuwkoop and Faber, 1994). Although the tadpole’s morphology is stable during this time, we determined that the observed green preference is still developing during the early days of stage 48 at 11–12 dpf but remains stable from 13 to 18 dpf (Supplementary Figure S1). To control for this slight heterogeneity within stages 48/49, all color preference experiments were performed on tadpoles that were 13–18 dpf, an age range in which the green preference is stable.

### Open-Field Group Color Preference Assay

In a naturalistic setting, animals are constantly interacting with and adjusting to their environments across time. An animal’s first motor behaviors in a novel environment do not necessarily reflect its motor behaviors across time. To account for this and quantify tadpole color preference over time, we developed a novel assay that utilizes a scan sampling methodology (Altman, 1974). Ten wild-type *Xenopus* tadpoles were transferred into a 140 mm-diameter circular petri dish filled with Steinberg’s solution to a depth of 6 mm (75 ml). The dish was surrounded with white paper to obscure any extraneous visual stimuli and placed on an LCD computer monitor (HP ZR22W or Dell E198FPf) so that light was projected onto the floor of the dish. Tadpoles were allowed to acclimate for 1 min and then their behavior was recorded using a digital video camera (GoPro, San Mateo, CA, USA) for subsequent analysis. To avoid potential circadian-dependent variations (oscillations) on visually guided behaviors, all color preference assays were run between 2:00 p.m. and 4:00 p.m.

**Figure 1:**
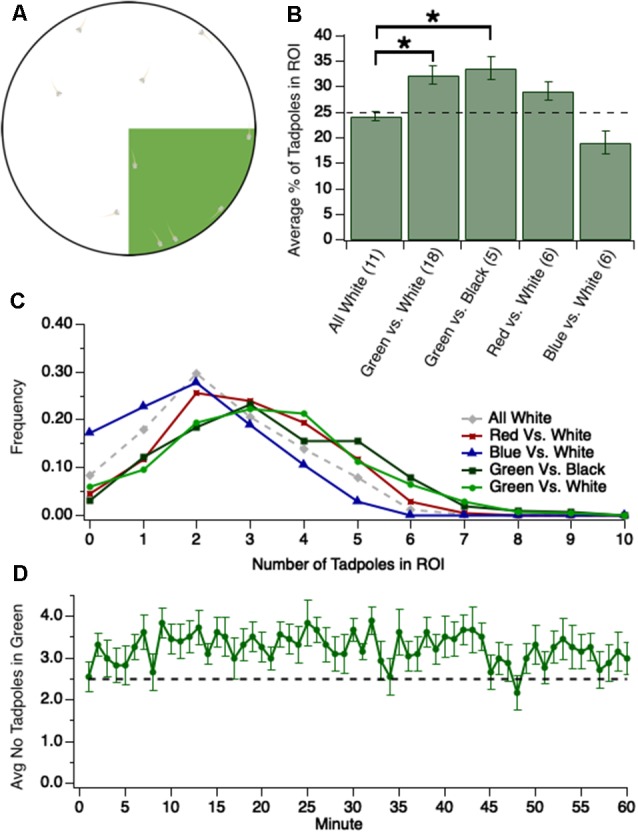
Tadpoles spend significantly more time in the green region of test dish when green is pitted against either white (brighter than green) or black (darker than green).** (A)** Experimental design: 1/4th of the floor of the test dish, the region of interest (ROI), is colored either green (shown here), blue, or red. The colors are approximately equiluminant. Ten tadpoles are transferred into the dish and videotaped for either 1 h or 30 min as they swim freely around the dish, and videos are analyzed off-line. **(B)** Bar graph showing the average number of tadpoles residing for different colored ROIs, pitted against black or white (*p < 0.05). The dashed line represents the percentage of tadpoles expected to be in the ROI if no preference. **(C)** Normalized frequency (Y-axis) of observing a given number of tadpoles in a given ROI (X-axis). **(D)** Plot showing the average number of tadpoles (out of 10 total) in the green quadrant at each minute, across 60 min. Notice that the average strength of preference for the green quadrant is modest yet stable over time, and is established by minute 2 of the trial. The trace/function represents 18 trials. The dashed line represents the average number of tadpoles expected to be in the green quadrant, at any given point in time, if no preference.

At every minute of the recording, on the minute, the number of tadpoles in the region of interest was counted to characterize the color preferences of tadpoles over time (Figure 1A). A tadpole was considered to be in the region of interest if both its eyes were in the region of interest. If one eye was in the region of interest and one eye not, it was then determined that the tadpole was in the region of interest only if a majority of the tadpole’s head was in the region of interest. Otherwise, the tadpole was deemed not to be in the region of interest.

The light projected onto the floor of the dish varied by experiment. Except for one control experiment in which the entire floor of the dish was white, the light was always projected such that one quadrant of the dish, comprising 25% of the dish’s floor, was one color and the other three quadrants were black or white. All visual stimuli were generated in Adobe Photoshop Creative Cloud 2018, version 19.1.4, in Lab color mode. Within Photoshop, the white stimulus had a Lightness Channel (L) value of 100 while the black stimulus had an L value of 0. The green stimulus had Lab values of L: 75, a: −75, b: 75 (closest RGB: 0, 216, 0). The blue stimulus had Lab values of L: 25, a: 50, b: −100 (closest RGB: 0, 33, 221). The red stimulus had Lab values of L: 50, a: 75, b: 50 (closest RGB: 232, 20, 42).

The initial experiments examining green preference in wild-type tadpoles were conducted by recording tadpole behavior for 60 min per trial. However, it was determined that preference does not vary over time (Figure 1D). Thus, subsequent experiments recorded tadpole behavior for 30 min per trial.

A green preference trial was excluded from analysis only if at least 75% of the tadpoles were immobile in the dish for at least 10 consecutive minutes of the recording. In such cases, the immobility of the tadpoles means that any analysis would no longer be quantifying tadpole swimming behaviors. This criterion excluded only two of 78 trials. Each group of 10 tadpoles was tested only once, except for the optic tectum (OT)-ablated and tegmentum-ablated groups, which were tested twice to minimize the number of tadpoles receiving ablations.

### Behavioral Dynamics

#### Individual Preferences

To assess the preferences of individual tadpoles within a group, individuals from two trials were manually tracked for 30 min. Using the same criteria, we employed in quantifying group preferences, at each minute time point, it was determined whether the individual was swimming in the green quadrant of the dish. In this way, we determined the percentage of time each tadpole spent in the green quadrant of the dish.

#### Swimming Speed

We quantified swimming speed for those trials in which the green preference was strongest by measuring the distance a tadpole traveled in the 300 frames (10 s) immediately after entering the green or a designated white quadrant of the dish. For each of the seven videos analyzed, the first five border crossings after the 15-min mark (the 27,000th frame of video) were recorded for the borders of both the green and designated white quadrants of the dish. All swimming distances were quantified in ImageJ, version 1.51m9 using the Measure function.

For inclusion in the data set, the tadpole had to remain in the area of interest (i.e., the green or white quadrant of the dish) for at least 150 of the 300 frames (5 of the 10 s). For example, if a tadpole entered the green quadrant of the dish but exited the green quadrant 30 frames (1 s) later, that data point could not be used for quantifying the swimming speed in the green. However, this was an exceedingly rare occurrence—this criterion excluded only 4 of the 39 trials.

#### Crossover Analysis

**Figure 2:**
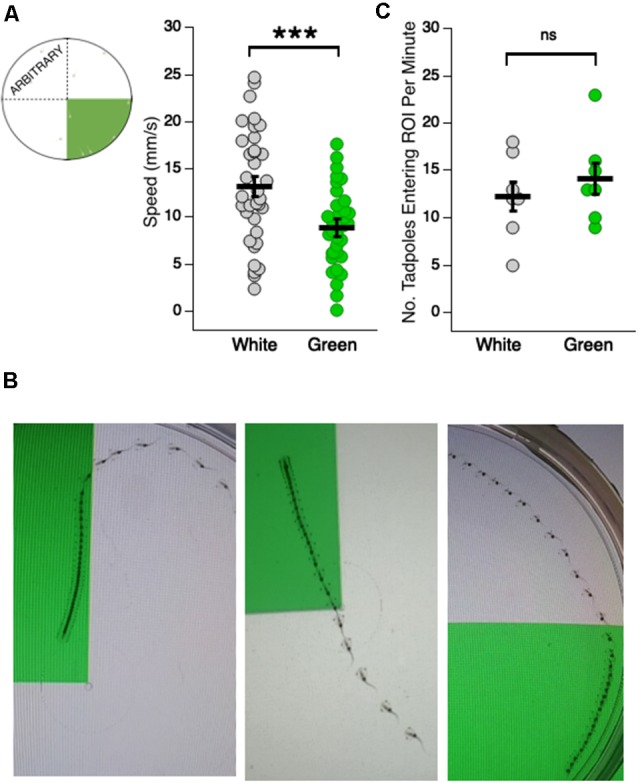
Tadpoles swim slower in the green region of the test dish. (**A**, far left) Schematic showing the arbitrary white quadrant used for quantifying swimming speeds in the white region; (right) dot plot of swimming speeds while in the white and green region of the test dish (***p < 0.001). Each dot represents the recorded speed of an individual tadpole. **(B)** Merged video frames showing (left and middle panels) single tadpoles slowing down upon entering the green region of the dish, and (right) a tadpole speeding upon swimming out of the green and into the white region. The faster the tadpole is swimming, the greater distance traveled between frames and visa versa. **(C)** Dot plot showing the number of times a given tadpole entered a designated white quadrant of the dish, and the number of times they entered the green quadrant (ns = not significant). The probability of freely-swimming tadpoles to enter the green quadrant of the dish is not statistically different from the probability of their moving into a designated white quadrant.

To quantify the frequency with which tadpoles crossed over into the green quadrant, we counted both the number of crossovers into the green quadrant and the number of crossovers into the quadrant directly opposite the green quadrant in the white section of the dish (Figure 2A schematic). All crossover measurements were conducted for the 10th minute of each video (frames 18,000–19,800).

### Dark-Rearing

To test whether a visual experience is necessary for the expression of color preference, some tadpoles were reared in the absence of all visual stimuli, i.e., complete darkness, from developmental stage 25 (approximately 3 dpf) until testing at stage 48/49 (13–18 dpf). At stage 25, *Xenopus* tadpoles have not yet formed an eye cup (Nieuwkoop and Faber, 1994). Thus, no visual information reached any brain regions before the beginning of the dark-rearing procedure.

As with control tadpoles, dark-reared tadpoles were reared in an incubator at 22°C. The incubator had no lights on the interior, but to account for any possibility of light leaking in, bowls were additionally covered entirely with aluminum foil. This aluminum foil remained in place over the bowls until seconds before testing began, minimizing exposure to any visual stimuli until color preference could be assessed.

### Tectal and Tegmental Ablations

For all ablation surgeries, stage 48 tadpoles were anesthetized in 0.01% MS-222 until they were no longer swimming (approximately 5–10 min). Once fully anesthetized, individual tadpoles were transferred onto a Steinberg’s solution-moistened paper towel. Using a sterile, 25-gauge needle, a small incision was made through the skin overlying the OT, at the midline, to access the OT and tegmentum.

#### Tectal Ablations

**Figure 3:**
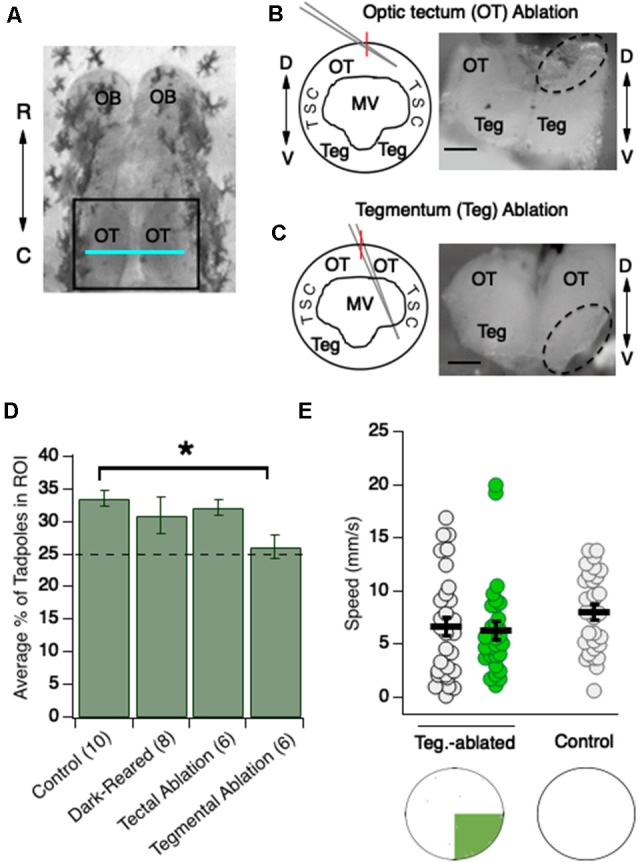
Ablation studies indicate that the preference for the green region of the dish requires the tegmentum but not the OT. **(A)** Overhead view of stage 48 tadpole brain. OT (region inside black square) is a major component of the amphibian midbrain; the blue horizontal line indicates the approximate position along the rostrocaudal axis at which the OT is most extensive. The tegmentum resides directly ventral to this region of the OT, and so is not visible in this image. OB, olfactory bulb; OT, optic tectum; R, rostral; C, caudal. (**B**, schematic) Cross-section of this region. The dorsal optic tectum and ventral tegmentum are separated in space by the large MV. For optic tectum ablations, the pipette is advanced at an oblique angle to ablate specifically the OT; (**B**, micrograph) cross-section showing an ablated tectal lobe. D, Dorsal; MV, Middle Ventricle; TSC, torus semicircularis; Teg, Tegmentum. (**C**, schematic) Cross-section showing that for tegmentum ablations, the pipette is advanced at a steeper angle. (**C**, micrograph) Cross-section showing an ablated tegmentum. The dashed-line-enclosed regions are the regions that have been ablated. Here, only one hemisphere is ablated to readily compare “normal” and “ablated” structures. For the experiments, both hemispheres are ablated. Red vertical lines on the schematics indicate the midline. Scale bar = 100 μm for both images. **(D)** Bar graph summarizing the effects of dark-rearing, tectal ablation, and tegmental ablation (*p < 0.05). Only the tegmental-ablated show a significant loss in preference for green compared to controls. **(E)** Dot plots showing swimming speeds of tegmentum-ablated tadpoles while in the white and green region of the test dish, and speeds of control tadpoles in an all-white dish. Each dot represents an individual tadpole’s swimming speed. Notice that average swimming speeds for tegmental-ablated tadpoles are similar across the green and white test dish, and are also similar to swimming speeds of control tadpoles in an all-white dish.

A sharp glass pipette controlled by a Leica Microsystems manipulator (Wetzlar, Germany) was advanced through the incision at an oblique, almost horizontal angle to access the dorsally situated lobe of the OT, contralateral to the side of the manipulator (Figure 3B). Similar to the procedure reported by McKeown et al. (2013), the pipette was advanced and retracted to create one or two stab wounds across the entire lobe of the OT, however, no aspiration was applied to remove neurons. Next, the pipette was completely retracted, the tadpole turned 180°, and the procedure repeated on the other OT lobe.

#### Tegmental Ablations

The tegmentum is situated directly ventral to the caudal, most extensive (“widest”) region of the OT (Figure 3A; Bernardini et al., 2010; Miraucourt et al., 2012; Bandin et al., 2013). The two midbrain regions are separated along the D-V axis by the large middle ventricle (MV; Figures 3B,C).

Tegmentum ablations were carried out similarly to tectal ablations except the pipette is positioned at a steeper angle such that it transverses the large MV to pierce the tegmentum (Figure 3C). The size of the incision through the overlying skin and the size of the needle was the same for the different ablation procedures, the only difference being the angle and distance by which the needle was advanced into the brain (Figures 3B,C schematic).

### Pharmacological Manipulations

For chronic exposure of tadpoles to the selective serotonin reuptake inhibitor (SSRI) trazodone, either 1 μM or 10 μM of trazodone hydrochloride (Sigma–Aldrich, catalog # T6154) was added to their Steinberg’s rearing solution from developmental stage 43 (approximately 4 dpf) until they were tested for color preferences (stage 48/49, approximately 13–18 dpf). The rearing solution for all groups was replaced every 48 h until completion of the experiment. To observe the acute effects of trazodone, tadpoles were exposed to the same concentrations only 24 h before testing. In both the acute and chronic experiments, 10 tadpoles from each group underwent 30-min trials on a green vs. white and green vs. black assay over 2 days.

### Statistical Analysis

All descriptive statistics are reported as mean ± standard error of the mean (SEM). Several data sets were determined to follow a non-normal distribution by Shapiro-Wilk test (observations for number of wild-type tadpoles in the green quadrant of a green-and-white dish: *W* = 0.962, *p* = 2e–16, *n* = 1,620; tegmental-ablated tadpole speed data, speed in white quadrant: *W* = 0.918, *p* = 0.02, *n* = 30, green quadrant: *W* = 0.821, *p* = 1e–4, *n* = 30). As such, non-parametric tests were used for all analyses. Kruskal–Wallis test was used for multiple-group comparisons and all pairwise comparisons were performed with a non-parametric Mann–Whitney U test, with the Benjamini and Hochberg (1995) correction for multiple comparisons as appropriate. Comparisons to theoretical values were performed using a Wilcoxon signed-rank test. Statistical analyses were carried out using R (R Core Team, 2020), Excel, and Graphpad/Prism.

## Results

For all experiments,10 stage 48/49 tadpoles (approximately 13–18 dpf) were placed in a large petri dish sitting atop a computer monitor. 1/4th of the petri dish was illuminated by a given color (green, blue, or red) and the remaining 3/4th of the dish was either white or black (Figure 1A). Tadpoles were videotaped for 30 min as they swam freely around the dish and the number of tadpoles in the colored quadrant of the dish was counted every minute. Given that the colored section of the dish is 1/4th the entire area, an average of 25% of the tadpoles would be predicted to occupy the colored quadrant at any given point in time if there was no preference or avoidance for a given color. Values greater or less than 25% would indicate a preference or avoidance of the colored quadrant, respectively. This theoretical value of 25% assumes a random distribution of tadpoles across the dish. To test for schooling behavior, which may create an uneven distribution of tadpoles across the dish, tadpoles were recorded in an all-white dish. We found that, on average, 24.4 ± 0.9% (*n* = 11 trials) of the tadpoles occupied a designated quadrant of the all-white dish at any given point in time (Figure 1B), reflecting an even distribution of tadpoles across the test dish. Furthermore, the two most common values for the number of tadpoles in the designated quadrant were 2 and 3, suggesting that no schooling occurred (Figure 1C).

### Tadpoles Display a Modest Yet Consistent Preference for Green Over White

It has previously been reported that stage 48 *Xenopus* tadpoles phototax towards light and away from dark (Moriya et al., 1996), but it has also been reported that, at this same developmental stage, they prefer green over other colors (Jaeger and Hailman, 1976). To determine which preference takes precedence, we pitted green against white. We observed that, compared to the designated quadrant of the all-white dish, significantly more tadpoles occupied the green quadrant at any given time [Figure 1B, Kruskal–Wallis test comparing all groups: *p* = 0.001, total *n* = 46 trials, individual group sample sizes reported below alongside groups’ respective pairwise comparisons; the average number of tadpoles in the designated quadrant for the all-white dish: 24.4 ± 0.9%, *n* = 11 trials; green quadrant: 32.4 ± 1.8%, *n* = 18 trials; Mann–Whitney U test *post hoc* comparison between the all-white dish and green and white dish, with Benjamini-Hochberg (BH) adjustment: *p* = 0.02]. Indeed, we most frequently observed 3 or 4 tadpoles in the green quadrant (Figure 1C). The modest nature of the preference for green may be due to the simultaneous, competing attraction to the more luminous white area of the dish. If this were the case, then the preference for green would be more pronounced when tested against black. The observed preference for green when pitted against black, however, was similar to that when pitted against white (Figure 1B, green and black dish: 33.6 ± 2.2%, *n* = 5 trials; comparison to the green and white dish (*n* = 18) *via* Mann–Whitney U test *post hoc* with BH adjustment: *p* = 0.85). The distribution of the number of tadpoles in the green quadrant was similar between both experiments (Figure 1C), suggesting that the preference for green is not dampened by a competing attraction towards greater luminance (white). This contrasts with previous work, which has reported that luminance preference may overshadow color preference during the tadpole’s *initial* phototactic decision (Jaeger and Hailman, 1976). In light of this difference in behavior, we investigated whether tadpoles, over time, display preferences for or against other colors in comparison to white. We found that tadpoles tended to spend more time in a red quadrant than white, albeit the preference for red was not statistically significant [Figure 1B; the average number of tadpoles in red quadrant: 29.1% ± 1.9%, *n* = 6 trials; comparison to the all-white dish (*n* = 11) *via* Mann–Whitney U test *post hoc* with BH adjustment: *p* = 0.11). Interestingly, tadpoles tended to avoid blue, though this too was not statistically significant (Figure 1B; the average number of tadpoles in blue quadrant: 19.1% ± 2.3%, *n* = 6 trials; comparison to the all-white dish (*n* = 11) *via* Mann–Whitney U test *post hoc* with BH adjustment: *p* = 0.11].

Overall, these data indicate that stage 48/49 tadpoles, when given a choice, will spend significantly more time in the green quadrant, regardless of whether the remaining 3/4th of the dish is black (darker than green) or white (brighter than green). The green preference over white data was further analyzed by plotting the number of tadpoles in the green quadrant at each minute of the trial. This plot, shown in Figure 1D, revealed that the preference for green is established by the first minute and that the degree of preference is stable throughout the entire trial. Thus, the preference for green is both consistent and persistent in degree not only across trials, but within trials as well.

### Preference for Green Displayed by Individual Tadpoles Closely Matches the Group Average

The observed preference for the green could be due to all tadpoles displaying a modest preference for green, or it could reflect another distribution, such as a bimodal distribution in which some of the tadpoles strongly prefer green and some avoid green. To determine which is the case, 10 individual tadpoles were tracked and for each individual, it was determined at each time point (1-min intervals) whether the tadpole was in the green quadrant. This analysis revealed that the preference displayed by individual tadpoles closely mirrors the overall group average. Indeed, an individual tadpole’s preference for green differed from the mean of the group with which it was tested by only 11% on average, with no individual tadpole’s green preference differing from the group mean by more than 19% (Table 1), indicating that the magnitude of the group preference reflects each tadpole’s preference.

**Table 1:** Preference for green displayed by individual tadpoles compared to the group preference.

Tadpole Group	1	2	3	4	5	6	7	8	9	10
	1	1	1	1	1	2	2	2	2	2
Group Mean Time in Green (%)	42.33	42.33	42.33	42.33	42.33	37.67	37.67	37.67	37.67	37.67
Individual Time in Green (%)	26.67	56.67	23.33	33.33	60	36.67	23.33	43.33	46.67	33.33
Difference from Group Mean (%)	15.67	14.33	19	9	17.67	1	14.33	5.67	9	4.33

### Changes in Swimming Speed Wholly Account for the Green Preference

A preference for green could arise due to a change in swimming behavior upon entering the green, an increased frequency with which tadpoles enter the green, or a combination of both. To assess changes in swimming behavior, the swimming speeds of individual tadpoles were measured while in the white and the green portion of the dish. We observed that tadpoles swam significantly slower in the green portion of the dish compared to the white portion (Figure 2A; average speed in white: 13.14 ± 1.14 mm/s, *n* = 35 tadpoles; average speed in green: 8.82 ± 0.92 mm/s, *n* = 35 tadpoles; Mann–Whitney U test: *p* = 2e–16). Indeed, tadpoles showed a strikingly rapid slow-down upon entering the green and oftentimes sped up as they moved out of the green and into the white. Illustrative examples of the differences in tadpole swimming speed between green and white sections of the dish are shown in Figure 2B. To determine whether an increase in the number of times tadpoles cross into the green quadrant also contributed to the green preference, we counted the number of times tadpoles crossed from the white to the green region of the dish over a 1 min time interval. We compared this result to the number of times a tadpole crossed into a designated (“arbitrary”) white quadrant (Figure 2C). There was no significant difference between the two groups (green quadrant: 14.14 ± 1.62 crosses/min, *n* = 7 min; white quadrant: 12.29 ± 1.6 crosses/min, *n* = 7 min; Mann–Whitney U test: *p* = 0.56). Taken together, these results indicate that changes in swimming behavior upon entering the green section of the dish can wholly account for the green preference. Specifically, tadpoles spend more time in the green because they slow down upon entering the green.

### The Preference for Green Is Experience-Independent

It is well established that, during the development of sensory circuits, sensory-driven experience provides the activity for activity-dependent mechanisms. To determine if the expression of innate color preference in these stage 48/49 *Xenopus* tadpoles requires visual experience during development, tadpoles were dark-reared (DR), in the absence of all visual stimuli, from developmental stage 25 (approximately 3 dpf, before the eyecup has formed) until stage 48/49 (approximately 13–18 dpf), and color preference-tested using the same assay depicted in Figure 1A. We found that the preference for green exhibited by DR tadpoles did not differ from that of wild-type tadpoles reared on a 12:12 light/dark schedule (Figure 3B, Kruskal-Wallis test comparing all groups: *p* = 0.03, total *n* = 30 trials, individual group sample sizes reported below alongside groups’ respective pairwise comparisons; the average number of DR tadpoles in green quadrant: 30.9 ± 2.8%, *n* = 8 trials; wild-type tadpoles: 33.5 ± 1.2%, *n* = 10; Mann–Whitney U test *post hoc* comparison between tectal-ablated and wild-type tadpoles, with BH adjustment: *p* = 0.62), indicating that the circuitry underlying the innate color preference develops independently of visual experience. Although the results here show that visually-driven activity is not necessary for color preferences, this does not rule out the possibility that spontaneous, sensory-independent activity could be important for the proper development of the underlying circuit(s).

### Ablating the Tegmentum Abolishes Color Preference

Next, we asked what visual circuits underlie the observed color preference behavior. While it has been determined that visual avoidance behavior in tadpoles requires a properly functioning OT (Dong et al., 2009; Shen et al., 2014; Liu et al., 2018), the brain regions involved in color perception have not been described. To determine if the OT is involved in color preference, tectal ablations were performed as described by (Figure 3B; Dong et al., 2009), typically between 12 and 18 h before testing. We observed that tectal-ablated tadpoles displayed the same degree of preference for green over white as control tadpoles. In other words, despite the tectum’s role in other visually-guided behaviors, the ablation of the tectum did not affect color preference (Figure 3D; avg. number of tectal-ablated tadpoles in green quadrant: 32.1 ± 1.3%, *n* = 6 trials; wild-type tadpoles: 33.5 ± 1.2%, *n* = 10 trials; Mann–Whitney U test *post hoc* comparison between tectal-ablated and wild-type tadpoles, with BH adjustment: *p* = 0.62).

Another candidate structure to underlie color preference is the tegmentum, a midbrain structure residing ventral to the widest (i.e., most extensive) region of the OT (Figure 3A). Although a role for the tegmentum in visual processing has not yet been described, anatomical studies describe retinal ganglion cell (RGC) axon input to the tegmentum (McDiarmid and Altig, 1999), and our whole-cell recordings from presumptive tegmental neurons indicate that they receive strong monosynaptic input from RGC axons (unpublished data). To test whether the tegmentum is necessary for color preference, it was surgically ablated. The size of the incision and the size of the needle were the same for the different ablation procedures, the only difference being the angle and distance by which the needle was advanced into the brain (compare Figures 3B,C schematic). Ablating the tegmentum eliminated the display of color preference (Figure 3D; average number of tegmental-ablated tadpoles in green quadrant: 26.2 ± 1.8%, *n* = 6 trials; Mann–Whitney U test *post hoc* comparison to wild-type tadpoles (*n* = 10), with BH adjustment: *p* = 0.03). Indeed, further analysis shows the amount of time spent by tegmentum-ablated tadpoles in the green quadrant did not differ from what would be expected by chance, behaving as if colorblind (Figure 3D; one-sample Wilcoxon signed-rank test comparing to 25%: *p* = 0.40). The absence of a behavioral preference suggests that the tegmentum is necessary for color perception and/or the color-guided behaviors observed in this study.

Given the lack of a green preference for tegmental-ablated tadpoles, and given that the green preference in normal tadpoles arises from changes in swimming speed upon entering the green, we predicted there would be no changes in swimming speed when tegmental-ablated tadpoles entered the green. To test this prediction, we analyzed swimming speeds in both the green and a designated (arbitrary) white quadrant for tegmental-ablated tadpoles, as carried out earlier in this study (Figure 2). We observed no overall difference in swimming speed between white and green quadrants (Figure 3E; avg. speed in white: 6.63 ± 0.89 mm/s, *n* = 30 tadpoles; average speed in green: 6.34 ± 0.79 mm/s, *n* = 30 tadpoles; Mann–Whitney U test: *p* = 0.99). This result shows that the behavioral mechanism which gives rise to green preference in normal tadpoles is eliminated with ablation of the tegmentum. It is important to note that while tegmentum-ablated tadpoles, on average, displayed consistent swimming speeds across the white and green regions of the test dish, their overall speed was also slower compared to control tadpoles, even slower than control tadpoles in the green region of the dish (Figure 2A). One explanation for this observation is that the ablation procedure may sever axons of either motor neurons of the tegmentum or other descending projections that pass by the tegmentum. Alternatively, it could be due to the inability of the tegmentum-ablated tadpoles to perceive the differing visual scene across the test dish. We reasoned that the visual scene in the test dish—a white and green region, and a border between the two—could be enhancing the motor activity of normal tadpoles. If this were true, it would be expected that normal tadpoles would display slower swimming speeds in a test dish that was uniform in color and luminance. To test this possibility, we measured swimming speeds of tadpoles in an all-white test dish. Average speeds in the all-white dish were observed to be noticeably slower compared to speeds recorded in the green and white test dish (Figure 3E; average speed of normal tadpoles in all-white test dish: 8.0 ± 0.68 mm/s, *n* = 30), and more similar to average speeds of the tegmentum-ablated tadpoles in the usual green and white test dish. This shows that that the colored pattern of the test dish itself stimulates motor activity, but not in tegmental-ablated tadpoles.

### Exposure to the Selective Serotonin Reuptake Inhibitor Trazodone Switches the Preference From Color-Based to Luminance-Based

**Figure 4:**
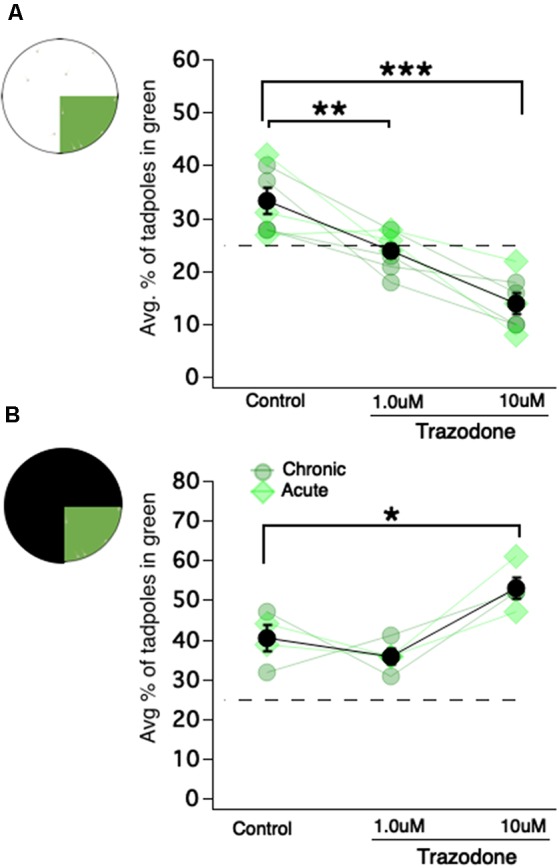
Exposing *Xenopus* tadpoles to the selective serotonin reuptake inhibitor (SSRI) trazodone switches the preference for the green region of the dish to the brightest region of the dish, regardless of color. **(A)** A schematic of the green vs. white test and corresponding dot plot showing the average percentage of tadpoles in the green region as a function of trazodone concentration (**p < 0.01, ***p < 0.001). Each connected set of symbols represents the average values for a given clutch. The overall average (shown in black) represents the data obtained from seven different clutches of tadpoles. Acute exposure to trazodone (light green diamonds) and chronic exposure (gray-ish green circles) appeared to elicit the same effect. **(B)** Four of the seven clutches tested in the green vs. white test (shown in panel **A**) were also tested in the green vs. black test (*p < 0.05). These data are also displayed as a dot plot showing the average percentage of tadpoles in the green region as a function of trazodone concentration.

It is well known that serotonergic neurons in the tadpole raphe nucleus (located in the hindbrain) modulate swimming behavior in young *Xenopus* tadpoles (Sillar et al., 1992; Wedderburn and Sillar, 1994; Demarque and Spitzer, 2010; Hachoumi and Sillar, 2019). Also, in zebrafish, serotonin (aka 5-hydroxytryptamine, aka 5HT) has been shown to modulate phototaxis (Burgess et al., 2010; Cheng et al., 2016). Based on these findings, we asked whether 5HT modulates the magnitude or valence of the observed color preferences. To test this, tadpoles were exposed to the SSRI trazodone (1 μM and 10 μM) chronically (starting at developmental stage 43, thus approximately 10 days before testing) or acutely (24 h before testing). Control and trazodone-exposed tadpoles were then tested on the same green vs. white and green vs. black preference tests as previously described. Since chronic and acute exposures to trazodone produced similar effects, the results of these two groups were combined. In the green vs. white test, control tadpoles displayed the same characteristic preference for the green quadrant of the dish, while both the 1 μM and 10 μM trazodone-exposed tadpoles switched their preference, spending most of their time in the white (brighter) portion of the dish (Figure 4A; Kruskal–Wallis test comparing all groups: *p* = 4e–4, total *n* = 21 trials, individual group sample sizes reported below; avg. % of tadpoles in green quadrant for control: 33.3 ± 2.4%, *n* = 7; for the 1 μM trazodone group: 24.0 ± 1.3%, *n* = 7; for the 10 μM trazodone group: 13.9 ± 1.9%, *n* = 7; Mann–Whitney U test *post hoc* comparisons with BH correction: *p* = 0.01 for control vs. 1 μM, *p* = 0.006 for control vs.10 μM trazodone). For the green vs. black test, the controls once again displayed a moderate preference for the green region of the dish, as did the 1 μM trazodone group, and the 10 μM trazodone group displayed an even stronger preference for the green (brighter) region of the dish (Figure 4B; avg. % of tadpoles in green quadrant observed for control: 40.35 ± 3.3%, *n* = 4; for the 1 μM trazodone group: 36 ± 1.9%, *n* = 4; for the 10 μM trazodone group: 52.8 ± 2.8%, *n* = 4; Mann–Whitney U test comparing control vs. 10 μM trazodone: 0.0294). To summarize, exposing tadpoles to the SSRI trazodone switched their behavior from preferring to reside in the green-colored region of the dish to whichever was the brighter (higher luminance) region. In other words, trazodone switched the tadpoles’ preference from color-based to luminance-based. As can be seen in Figure 4, both the chronic and acute exposures to trazodone produced similar effects, indicating that the observed switch from color-based to luminance-based preference was due to the acute effect(s) of the drug, most likely the modulation of serotonin transmission, and not to an alteration in developmental processes such as a change in the number of specific types of neurons or the way the circuit is wired. The acute switch from a color-based preference to one based more on luminance suggests that endogenous 5HT levels may govern the tadpoles’ visually-guided preferences.

## Discussion

The main findings of this study are that: (1) given a choice, freely swimming stage 48/49 tadpoles spend a significantly greater amount of time in the green region of the test dish when green is pitted against either white or black. Although modest in magnitude, this innate behavior is strikingly persistent over time and consistent across trials. (2) Relatively slower swimming speeds while in the green region of the test dish can fully account for the magnitude of the preference. (3) The results of the ablation experiments reveal that expression of the innate color preference requires the tegmentum but not the OT, suggesting two distinct visual streams in the tadpole and, to date, the first report of a role for the amphibian tegmentum in a color-guided behavior. (4) Exposure to the SSRI trazodone activates a preference for light that trumps preference for color, suggesting that the streams may be differentially modulated by 5HT.

### The Preference for Green Is Persistent Across Time

In this study, a novel open-field experimental paradigm was employed to determine innate, enduring color preferences of freely swimming *X. laevis* tadpoles. We found that tadpoles showed the strongest preference for the color green, a weak tendency to prefer red, and a weak tendency to avoid blue. These results are following previous phototactic studies of *Xenopus laevis* and *Rana temporaria*, which show that tadpoles at the same developmental stages tested in our study phototax most robustly towards green and away from blue (Muntz, 1963; Jaeger and Hailman, 1976). The experimental design used here in our study shows further that the innate preference for green is persistent over time, and that it is manifested mainly by a significant decrease in swimming speed in the green region of the dish—a behavior that we termed “photostasis.” Thus, mid-spectrum wavelengths of light (“green”) induce a decrease in motor activity. What may be the mechanism by which green visual stimuli cause tadpoles to swim slower? Based on work from the Spitzer lab (Demarque and Spitzer, 2010) showing that experimentally increasing the number of serotonergic neurons in the *Xenopus* tadpole raphe shortens swimming bouts, we hypothesized that perhaps activation of the visual system by medium wavelengths of light (“green”) may activate serotonin release in the raphe which in turn would depress motor activity. We reasoned that if this were the mechanism underlying the green-dependent decrease in motor activity, then experimentally enhancing endogenous 5HT transmission *via* a selective 5HT reuptake inhibitor would be expected to enhance the green-induced decrease in locomotion, resulting in a greater “preference” for green. The results did not support our hypothesis: trazodone-exposed tadpoles chose to reside in whichever region was the brightest (when green was pitted against white, they spent most of their time in the white region; when green was pitted against black, they preferred the green region). The presence of a wavelength-dependent component underlying this result cannot be ruled out, as the experimental paradigm used in this study does not test for preferences based purely on luminance. Because the motivation for testing the effect of an SSRI on the observed preference for green was to identify the mechanism that causes tadpoles to slow down when swimming in the green region of the dish, and not to study luminance preferences, the unexpected results are currently being further characterized in a separate study. Although the results of this experiment, fail to elucidate how a green visual stimulus can decrease locomotion, they do suggest that 5HT levels may dictate which qualities of a visual scene (color, brightness) the tadpoles respond to most. Furthermore, this is the first report of serotonin modulation of a visually guided behavior in tadpoles, and it is quite similar to a previous finding that exposing zebrafish to the SSRI fluoxetine increases phototaxis towards the light (Burgess et al., 2010; Cheng et al., 2016).

### Innate Color Preference Requires the Tegmentum

It has long been known that young *Xenopus* tadpoles can discriminate between different colors. Previous phototactic studies report innate color preferences and aversions displayed by tadpoles (Jaeger and Hailman, 1976), and associative learning paradigms are based on the ability of these tadpoles to readily discriminate between red and blue wavelengths of light (Blackiston and Levin, 2012; Rothman et al., 2016). Still, precisely how and where color stimuli are processed in the tadpole brain is unclear. The main component of the tadpole visual system is the retinotectal circuit. This circuit consists of the RGCs in the eye, which project their axons directly to the contralateral OT where they form synapses onto tectal neurons. Previous OT ablation studies have determined that the OT is required for visual avoidance behavior (Dong et al., 2009; McKeown et al., 2013), but is not required for the OMR, the tendency for tadpoles to follow moving bars of light (Dong et al., 2009). Here, by testing innate color preferences of OT- and tegmentum-ablated tadpoles, we identified another visual behavior that does not appear to require the OT, but that does require the tegmentum. While our results suggest an essential role for the tegmentum in an innate color-driven behavior, it does not rule out the possibility that the observed loss of color preference in tegmentum-ablated tadpoles could be due, instead, to disruption of other visually-guided swimming behaviors such as the OMR. This is unlikely to be the case, however, because the OMR is known to be associated with moving stimuli, while phototaxis is associated with stationary stimuli. Zebrafish are colorblind to moving bars of green and red light (Orger and Baier, 2005), suggesting that color-driven phototaxis and the OMR, at least in zebrafish, are functionally distinct behaviors governed by separate visual pathways. Moreover, our preliminary data indicate that tegmentum-ablated tadpoles display a normal OMR, indicating that the OMR is both an OT- and tegmentum-independent behavior, and, importantly, that the loss of color preference and overall slower swimming speeds of tegmental-ablated tadpoles is not due to impaired OMR. Overall, these data suggest that color-driven behavior in the amphibian requires the tegmentum, and further, that the tadpole visual system is comprised of a dorsal stream involving the OT and ventral stream involving the tegmentum. The tegmentum consists of several distinct nuclei which, in general, are thought to be comprised of motoneurons and interneurons. This suggests that the output from the tegmentum is motor-related. Functional inputs to the tegmentum have not been determined in the tadpole, although an anatomical study describes RGC axonal projections to the optic nucleus of the tegmentum (McDiarmid and Altig, 1999). In zebrafish, projections from the pretectum region to a defined set of neurons in the tegmentum have been shown to control visually-guided prey capture (Gahtan et al., 2005). Similarly, our tegmentum-ablation data suggest a role for the amphibian tegmentum in linking visual, color-driven input with a motor output.

### Shell Game Model Predicts a Modest Preference for Safety/Feeding Zones

The tadpoles used in this study displayed a moderate yet significant preference for the color green without ever being exposed to colors before testing. We found that tadpoles raised in complete darkness displayed the same degree of preference for green as those raised on a normal 12:12 light/dark schedule. Thus, the color-driven behavior described here is truly innate, and the underlying circuit, hardwired. In general, it is thought that innate behaviors work to increase the survival of the individual or population. Consistent with this idea, the innate tendency to phototax towards green has been suggested to improve the chances of survival by guiding the tadpoles to green aquatic plants, which provide their food and shelter (Muntz, 1963; Jaeger and Hailman, 1976). Similarly, the consistent preference for green that we observe here would lead to tadpoles spending most of their time in this region associated with food and safety, also promoting survival.

*Xenopus* tadpoles first begin foraging for food at stage 46 (McKeown et al., 2017), shortly before the developmental stages observed in this study. The development of feeding behaviors at this stage is consistent with the proposal that a preference for green may be associated with foraging and shelter-seeking. We found that tadpoles’ green preference is present under nutritionally restricted conditions, consistent with the report that neither OMRs nor visual avoidance behaviors are influenced by nutritional deprivation (McKeown et al., 2017). Together, these results paint a picture in which, even under conditions of food scarcity, hard-wired visually-guided behaviors are still expressed by *Xenopus* tadpoles, thereby promoting the chances of tadpoles’ survival.

But if residing in the green region truly increases chances of survival, why is the observed preference for green not more robust? It seems that if the green represents a source of food and safety, tadpoles would display a stronger preference. One possibility is that the green-colored floor, while indeed green, is not a plant and so does not evoke the optimal behavioral response. A more intriguing possibility, however, is that the consistently modest preference maximizes survival because if tadpoles resided in the safety/food source of green (plants) for relatively longer bouts of time, then predators would be more likely to learn the tadpoles’ (their prey’s) location. This strategy of a given prey spending only moderate amounts of time in a safe and/or feeding zone to be unpredictable to a given predator is a model of predator-prey dynamics referred to as the shell game (Mitchell and Lima, 2002). Although we do not determine unequivocally if the consistently moderate preference for the color green is a strategy that maximizes survival, considering the incredibly invasive capabilities of this particular amphibian and their vast evolutionary history (Furman et al., 2015) these tadpoles could be born masters of the shell game.

## Data Availability Statement

All datasets generated for this study are included in the article/Supplementary Material.

## Ethics Statement

All experiments involving animals were reviewed and approved by University of Wyoming Institutional Animal Care and Use Committee.

## Author Contributions

JH, JB, and KP designed, carried out, and analyzed the experiments. JH and KP wrote the manuscript and JB helped with the drafting.

## Conflict of Interest

The authors declare that the research was conducted in the absence of any commercial or financial relationships that could be construed as a potential conflict of interest.

## Supplementary Material

FIGURE S1 The green preference is stable from 13-18 dpf. Each marker represents the average green preference for one 60-min trial.
