# Supplementary figures and images for: An Innate Color Preference Displayed by Xenopus Tadpoles Is Persistent and Requires the Tegmentum
Source: Front Behav Neurosci. 2020 May 12;14:71. doi: 10.3389/fnbeh.2020.00071 (PMC7235192; doi:10.3389/fnbeh.2020.00071)

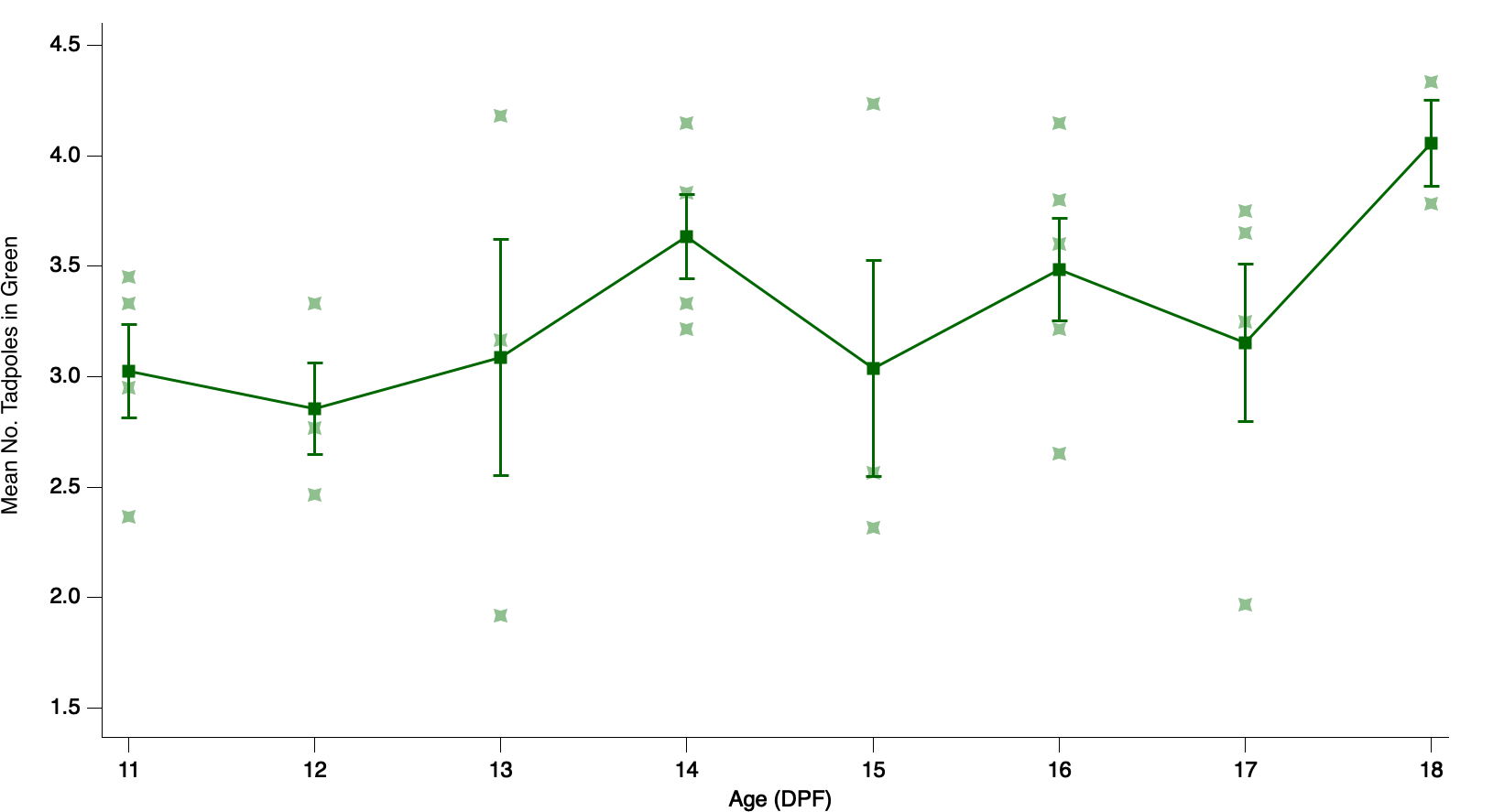

Supplement: FIGURE S1 — The green preference is stable from 13-18 dpf. Each marker represents the average green preference for one 60-min trial. [file Image_1.TIF]
